# Infection sequence alters disease severity—Effects of the sequential exposure of two larval trematodes to *Polypedates cruciger* tadpoles

**DOI:** 10.1002/ece3.5180

**Published:** 2019-04-26

**Authors:** Nuwandi U. K. Pathirana, Madhava Meegaskumbura, Rupika S. Rajakaruna

**Affiliations:** ^1^ Department of Zoology University of Peradeniya Peradeniya Sri Lanka; ^2^ Postgraduate Institute of Science University of Peradeniya Peradeniya Sri Lanka; ^3^ Freshwater Fish Group and Fish Health Unit, Centre for Sustainable Aquatic Ecosystems, School of Veterinary & Life Sciences Murdoch University Perth Australia; ^4^ Guangxi Key Laboratory of Forest Ecology & Conservation, College of Forestry Guangxi University Nanning China; ^5^ Department of Molecular Biology and Bio‐technology University of Peradeniya Peradeniya Sri Lanka

**Keywords:** *Acanthostomum burminis*, cercaria, frogs, furcocercous, trematodes

## Abstract

Multiple pathogens coexist in nature, and hence, host species often encounter several pathogens simultaneously. The sequence in which the host encounters the parasites influences interactions between parasites and host pathology. Here, the effects of infection by two cercaria (larvae of trematodes) types, pleurolophocercous cercaria of *Acanthostomum burminis* and a furcocercous cercaria, on the tadpoles of common hourglass tree frog (*Polypedates cruciger*) were examined. Ten days posthatch, tadpoles (Gosner stage 27/28) were used for infection exposures. First, in a single infection each cercaria type was introduced to the tadpoles separately. Second, coinfection of the two cercaria was carried out by alternating the sequences of exposure. For all the experiments, appropriate controls were instituted. Tadpoles of all groups exposed to parasites had lower survival levels compared to controls. Among the four groups exposed, the highest survival was observed in the coinfection when furcocercous was introduced first (82.5%). The lowest survival was observed in the coinfection when the *A. burminis* cercaria was introduced first (65.0%). In the coinfections, when *A. burminis* was introduced prior to furcocercous, survival of the tadpoles was reduced by 17.0% compared to the exposures of furcocercous prior to *A. burminis*. Prior infection with *A. burminis* induced negative effect on the host with an increased infection severity, while prior infection with furcocercous had reduced infection severity than lone exposures. These results suggest that furcocercous infections can be beneficial for hosts challenged with *A. burminis* provided that *A. burminis* exposure occurs second. None of the treatments had an effect on the growth of the tadpoles, but lengthening of developmental period was observed in some exposures. All exposed tadpoles developed malformations which were exclusively axial—kyphosis and scoliosis. However, there was no difference in the number of malformed individuals in the single infection (19.0%–25.0%) compared to coinfection (20.0%–22.5%) or between coinfections. The results suggest that the sequence of parasite exposure affects host–parasite interactions and hence the disease outcomes. Understanding the effects of coinfection on disease outcomes for hosts provides insight into disease dynamics.

## 1 INTRODUCTION

Hosts encounter multiple pathogens in nature, increasing the likelihood of coinfection, a condition where two pathogens exploit a host species one after the other (Hoverman, Mihaljevic, Richgels, Kerby, & Johnson, 2012; Pedersen & Babayan, 2011). Coinfecting pathogens interact within a host to influence host pathology, parasite transmission, and the evolution of virulence (Cox, 2001; Lively, 2009; López‐Villavicencio et al., 2011; Pedersen & Fenton, 2007; Telfer et al., 2008, 2010). Concurrent infections with multiple pathogens can have an effect on fitness consequences for the host (Bentwich et al., 1999; Jolles, Ezenwa, Etienne, Turner, & Olff, 2008; Petney & Andrews, 1998; Romansic et al., 2011).

The sequence in which hosts encounter parasites influences the interactions between parasites, and these priority effects have been identified in many systems (Alford & Wilbur, 1985; Connell & Slatyer, 1977; Hoverman, Hoye, & Johnson, 2013). Host immune systems could react differently, depending on the sequences of two parasites encountering a host (Abbas, Murphy, & Sher, 1996; Cox, 2001). Depending on the characteristics of the specific parasites involved, indirect interactions can be either positive (due to immune suppression) or negative (as a result of cross‐reactive immune responses; Pedersen & Fenton, 2007; Cobey & Lipsitch, 2013). Recent studies have demonstrated priority effects on the outcome of parasite interactions in hosts during coinfection by multiple pathogens (Hoverman et al., 2013; Jackson, Pleass, Cable, Bradley, & Tinsley, 2006; Karvonen, Seppälä, & Tellervo Valtonen, 2009; Leung & Poulin, 2011).

Coinfections of multiple parasites are common in amphibian hosts (Hoverman et al., 2012; Johnson & Buller, 2011). Many authors have highlighted the possibility of the interactive effects of multiple pathogens which may contribute to the worldwide decline of amphibian populations (Cunningham et al., 1996; Daszak, Cunningham, & Hyatt, 2003; Green, Converse, & Schrader, 2002; Greer, Berrill, & Wilson, 2005; Worthylake & Hovingh, 1989) and the abiotic environmental stressors can strongly add on to the disease severity in amphibians (Rohr et al., 2008). Highly virulent pathogens like ranaviruses, chytrid fungus, and macroparasites like trematodes (e.g., *Ribeiroia ondatrae* and echinostomes) co‐occur in amphibian communities (Hoverman et al., 2012; Huffman & Fried, 2012; Warne, LaBumbard, LaGrange, Vredenburg, & Catenazzi, 2016). Few studies have been experimentally tested the effects of co‐occurrence of these pathogens in amphibians (Hoverman et al., 2013; Romansic et al., 2011).

Trematode infections of amphibians have attracted substantial attention because they are also associated with mortality and deformities and are thought to be common at present than was historically recorded (Johnson, Lunde, Ritchie, & Launer, 1999; Rohr, Raffel, & Hall, 2010). Free swimming larval stage or the cercaria of trematodes emerge from the infected snail and actively penetrate tadpoles and encyst within frog tissues as metacercaria. Encysted metacercaria induce malformations, primarily affecting the limbs (Johnson et al., 1999; Koprivnikar et al., 2012; Rajakaruna, Piyatissa, Jayawardena, Navaratne, & Amerasinghe, 2008). During multiple infections of trematodes, interactions between parasites are influenced by the order in which the host encounters each parasite. The effect of *Ribeiroia* infection was reduced when the frogs were already infected with *Echinostoma*, but the reverse sequences had no effect (Hoverman et al., 2013). This is the only study where sequential exposure effects of trematode parasites have been shown for a frog species.

Pleurolophocercous cercaria of *Acanthostomum burminis,* a trematode that infects water snakes, induces malformations, reduces mortality, and affects the growth in the tadpoles of the common hourglass tree frog, *Polypedates cruciger* (Jayawardena, Rajakaruna, Navaratne, & Amerasinghe, 2010b). A recent study shows that the older tadpoles of *P. cruciger* show higher tolerance and resistance to parasitism by the same trematode species than younger tadpoles (Pathirana, Meegaskumbura, & Rajakaruna, 2016, 2017). The present study integrates the exposure sequence effect into amphibian–trematode system where two cercaria types, pleurolophocercous cercaria of *A. burminis* and furcocercous cercaria, were exposed to *P. cruciger* tadpoles. We investigated the differential infection severity, in terms of survival and malformations associated with sequence of exposure of tadpoles to the two parasites.

## 2 MATERIALS AND METHODS

### 2.1 Amphibian host species

The common hourglass tree frog (*P. cruciger*, Family Rhacophoridae) is a widely distributed endemic amphibian found in the wet, intermediate, and dry zones of Sri Lanka up to 1525 m (Manamendra‐Arachci & Dutta, 1996). This arboreal species inhabits secondary forests and anthropogenic habitats, and its populations are stable; hence, its threat status is considered Least Concern (LC; IUCN, 2018). Its foamy egg masses are attached to vegetation overhanging pools and ponds. These eggs hatch about 5 days following laying, and the tadpoles fall into water body underneath (Manamendra‐Arachci & Dutta, 1996).

### 2.2 Collection of *Polypedates cruciger* eggs

Foamy egg masses were hand‐collected, in April 2016, from ponds in the Royal Botanical Gardens at Peradeniya (7°16′16″N, 80°35′44″E) and brought to the laboratory in a cooler. These were hung over a water‐filled glass container for hatching to take place. After hatching, the tadpoles were transferred into glass tanks (15 × 15 × 25 cm) containing 1 L of dechlorinated tap water (~100 ml per tadpole) at a density of 10 tadpoles per tank.

### 2.3 Collection of cercaria from snails

Two species of freshwater snails, *Melanoides tuberculata* and *Mieniplotia scabra*, were collected from Hingula Oya (7°14′56.3°N 80°28′29.7°E) and Maha Oya (7°15′1242″N 80°26′47615″E) in Kegalle District, Sri Lanka, during May 2016. These species of freshwater snails co‐occur in the same habitat where the tadpoles of this frog species are found. Snails were placed in glass jars (~500 ml) filled with water from the same habitat and were brought to the laboratory in a cooler. Each snail was kept separately in a plastic cup containing 25 ml of water. These cups containing snails were placed near a window to receive sunlight for about 4 hr to induce shedding of cercaria, or exposed to an artificial light when sunlight was low. This setup was left at room temperature (27–30°C), and the water in the cup was removed daily to maintain a water quality conducive to the snails. Snails were observed under a dissecting microscope several times a day for shedding of cercaria.

Cercaria were isolated from the snails, and few cercaria were slide‐mounted using Gilson's fixative and borax carmine single stain, and were identified using morphology as described in Jayawardena, Rajakaruna, and Amerasinghe (2010a).

### 2.4 Exposures of tadpoles to cercaria

**Figure 1:**
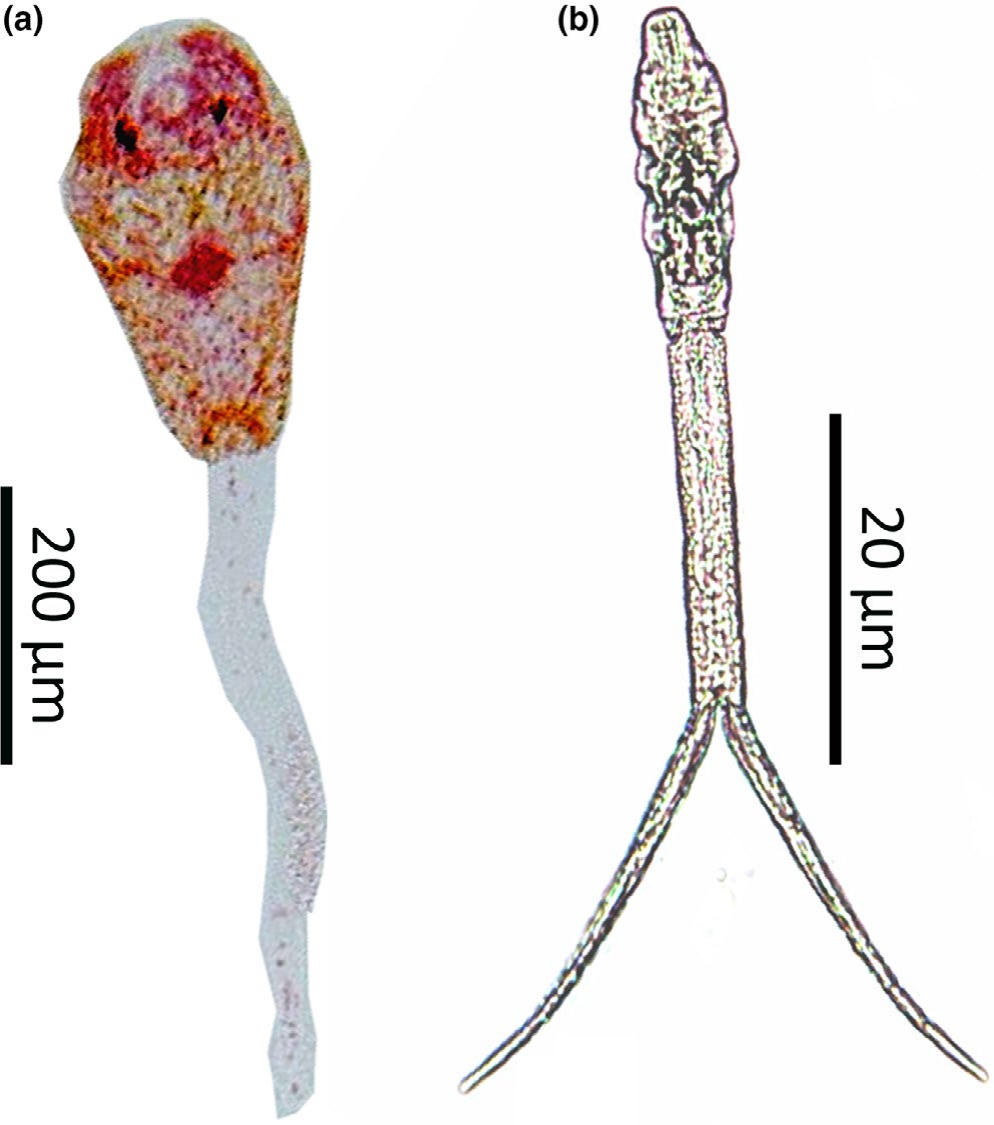
Two types of cercaria used to infect the tadpoles of *Polypedates cruciger* (a) Pleurolophocercous cercaria of *Acanthostomum burminis* isolated from *Melanoides tuberculata*; (b) furcocercous cercaria isolated from *Mieniplotia scabra*.

Ten days posthatch, tadpoles (Gosner stage 27/28) of *P. cruciger* were exposed to the two types of cercaria, *A. burminis* and furcocercous (Figure 1), in four treatments and a control (Table 1). Exposure of tadpoles to cercaria was conducted during May 2016. First, the lone exposures were carried out with the two types of cercaria by exposing each tadpole to 12 cercaria. Then, combined exposures were carried out with six cercaria from each type and exposing each tadpole to 12 cercaria. The second cercaria were introduced 30 min after introducing the first cercaria. A total of 200 tadpoles were tested in the study with 40 tadpoles per each treatment and 40 tadpoles as controls (in the absence of cercaria). Following the exposure to cercaria, tadpoles were raised individually until metamorphosis.

**Table 1:** Five exposure treatments used in the experiment with pleurolophocercous cercaria of *Acanthostomum burminis* (cercaria P) and furcocercous cercaria (cercaria F)

No.	Cercaria P	Cercaria F	Exposure sequence
1	−	−	Overall control
2	+	−	Cercaria P only
3	−	+	Cercaria F only
4	+	+	Both types. Cercaria P were exposed first
5	+	+	Both types. Cercaria B were exposed first

### 2.5 Counting metacercarial cysts

Metacercarial cysts in tadpoles were counted for five consecutive days following the procedure outlined in Rajakaruna et al. (2008). For this, ten tadpoles from each treatment were randomly selected and the number of cysts in each tadpole was counted. It may or may not be the same tadpole each time, but the mean of the counts for a treatment was considered. During counting of cysts, the tadpole was placed on a solid watch glass containing 20 ml of water and was examined under a dissecting microscope. The observations were carried out without anesthesia because the movement of the tadpole on the watch glass was limited and did not obstruct the counting. The metacercaria cysts were visible through the transparent skin of the tadpoles, and hence, the number of cysts and their position in the body were recorded. Ten tadpoles from each treatment were examined per day and returned to the original tanks after observation.

### 2.6 Laboratory rearing of the exposed tadpoles

All the exposed tadpoles were transferred into separate glass tanks (measuring 15 × 15 × 25 cm) containing ~200 ml of dechlorinated tap water and were raised individually until metamorphosis. The feces and debris collected at the bottom of the tank were siphoned out daily, and the water levels were replenished in order to maintain water quality; water in the tanks was completely replaced once a week. Tadpoles were fed with a commercial fish food, twice a day.

### 2.7 Collection of data

Survival of the tadpoles was recorded daily. The growth of tadpoles in each experimental and control setup was assessed at metamorphosis by measuring the snout to vent length (SVL), body mass, time to metamorphosis, and malformations. Snout to vent length was measured to the nearest 0.01 cm using a vernier caliper. Body weights were measured to the nearest 0.001 g using an electronic balance. Time required for forelimb emergence of half of the number of tadpoles (TE_50_) was recorded. Malformations were observed at 40 days posthatch (Gosner stage 31/32) and at metamorphosis. The malformations in tadpoles and metamorphs were categorized according to the “Field guide to malformations of frogs and toads” (Meteyer, 2000).

The study protocol was approved by the Ethical Review Committee of Postgraduate Institute of Science, University of Peradeniya, Sri Lanka. At the end of the study, all the exposed tadpoles and metamorphs and those in the control set up were euthanized using MS222 and preserved in 5% formalin.

### 2.8 Statistical analysis

Survival of the tadpoles in the exposed and control groups and the percentage malformations among different treatments were compared using binomial generalized linear model (GLM). The differences in the number of cysts were analyzed using one‐way ANOVA, and individual comparisons were done using Tukey's pairwise comparisons. The differences in growth parameters (SVL and body mass) and TE_50_ were analyzed using one‐way ANOVA, and individual comparisons were done using Dunnett's post hoc test. For survival data and growth parameters, the comparisons were first done with the control and treatments and then among the four treatments. For malformation data, comparisons were done among the treatments. MINITAB 18.0 for Windows was used for statistical analyses.

## 3 RESULTS

### 3.1 Shedding of cercaria

A total of 70 freshwater snails from each species were collected, of which 21 *M. tuberculata* (30.0%) shed *A. burminis* cercaria and 18 *M. scabra *(26.0%) shed furcocercous cercaria. The cercaria of *A. burminis* (Figure 1a) were slender and oval‐shaped transparent with a light brown color body (~250 µm) with a long and slender tail (~250 µm) which was approximately same length as the body. Oral sucker was prominent. Two eye spots can be found in the anterior part of the body. A pair of lateral fins in the anterior part of the tail was observed in live specimens. The cercaria first floated near the surface and later fell to the bottom of the cup. Furcocercous cercaria (Figure 1b) had elongated and transparent body (~100 µm) with a forked tail. Dark color penetration glands were located in the anterior region and were visible through the transparent body. Ventral sucker was smaller than the oral sucker. Tail stem (~40 µm) ended with comparatively longer furcae (~25 µm).

### 3.2 Lone exposures of tadpoles to cercaria

Furcocercous cercaria and the cercaria of *A. burminis* successfully penetrated the tadpoles of *P. cruciger* and encysted. This provides first empirical evidence of a furcocercous cercaria penetration and encystment in tadpoles although the encystment of cercaria of *A. burminis* in tadpoles of *P. cruciger* has been shown previously (Jayawardena, Rajakaruna, Navaratne, et al., 2010b). Exposure significantly reduced the survival in both groups (*A. burminis = *67.5%, binomial GLM, *F* = 9.47, *p* = 0.003, furcocercous = 75.0%, binomial GLM, *F* = 5.38, *p* = 0.023) compared to the control (95%). When the survival of the two lone exposures was compared, there was no difference in the reduction of survival of *P. cruciger *(*A. burminis* = 67.5% and furcocercous = 75.0%; binomial GLM; *F* = 0.54, *p* = 0.465; Table 2). Infection also induced malformations (*A. burminis* = 57.5% at 40 days posthatch and 25.0% at metamorphosis, furcocercous = 48.0% at 40 days posthatch and 19.0% at metamorphosis).

**Table 2:** Survival of tadpoles of *Polypedates cruciger* infected with pleurolophocercous cercaria of *Acanthostomum burminis* (cercaria P) and furcocercous (cercaria F) cercaria in the lone and combined exposures using binomial generalized linear model (GLM) test

		Comparison with overall control		Comparison with cercaria P only		Comparison with cercaria F only		Comparison with cercaria P + F		Comparison with cercaria F + P	
Treatment	Survival (%)	*F*	*p*	*F*	*p*	*F*	*p*	*F*	*p*	*F*	*p*
Cercaria P only	67.5	9.47	0.003**	–	–	0.54	0.465	0.05	0.816	3.44	0.067
Cercaria F only	75.0	5.38	0.023**	0.54	0.465	–	–	0.94	0.335	1.24	0.269
Cercaria P + Cercaria F	65.0	11.05	0.001*	0.05	0.816	0.94	0.335	–	–	4.39	0.039**
Cercaria F + Cercaria P	82.5	2.23	0.140	3.44	0.067	1.24	0.269	4.39	0.039**	–	–

Survival in the control = 95%.

* Significant differences at *p* < 0.001.

** Significant differences at *p* < 0.05, analyzed using GLM test, and the rest were not significant compared to the control (*n* = 40 tadpoles in each treatment and 40 in the control).

Malformations were scoliosis and kyphosis and were observed only in the tail region. Infection did not have a significant effect on the growth (measured as SVL and body weight) and the length of the developmental period of the tadpoles exposed to furcocercous (SVL, one‐way ANOVA; *F* = 0.53, *p* = 0.716; weight, one‐way ANOVA; *F* = 0.26, *p* = 0.903; TE_50_, one‐way ANOVA; *F* = 4.37, *p* = 0.285). However, a significant difference in the developmental period was observed in the tadpoles exposed to *A. burminis* (TE_50_, one‐way ANOVA; *F* = 4.37, *p* = 0.019).

### 3.3 Coinfection of the two parasites

#### 3.3.1 Survival

A significant reduction of survival was observed when the tadpoles were exposed to cercaria of *A. burminis* prior to furcocercous but not vice versa (binomial GLM, Table 2). The lowest survival and the highest survival were observed in the two combined exposures (*A. burminis* + furcocercous = 65.0% and furcocercous + *A. burminis* = 82.5%), and there was a significant difference between the two combined exposures (binomial GLM; *F* = 4.39, *p *= 0.039; Table 2). Infection of *A. burminis* followed by furcocercous seems to have an additive effect further reducing the lone effects on survival. When the infection sequence was reversed, it seems to have antagonistic effect, increasing the survival in the combined exposure more than the lone effects.

### 3.4 Parasitic cysts

**Figure 2:**
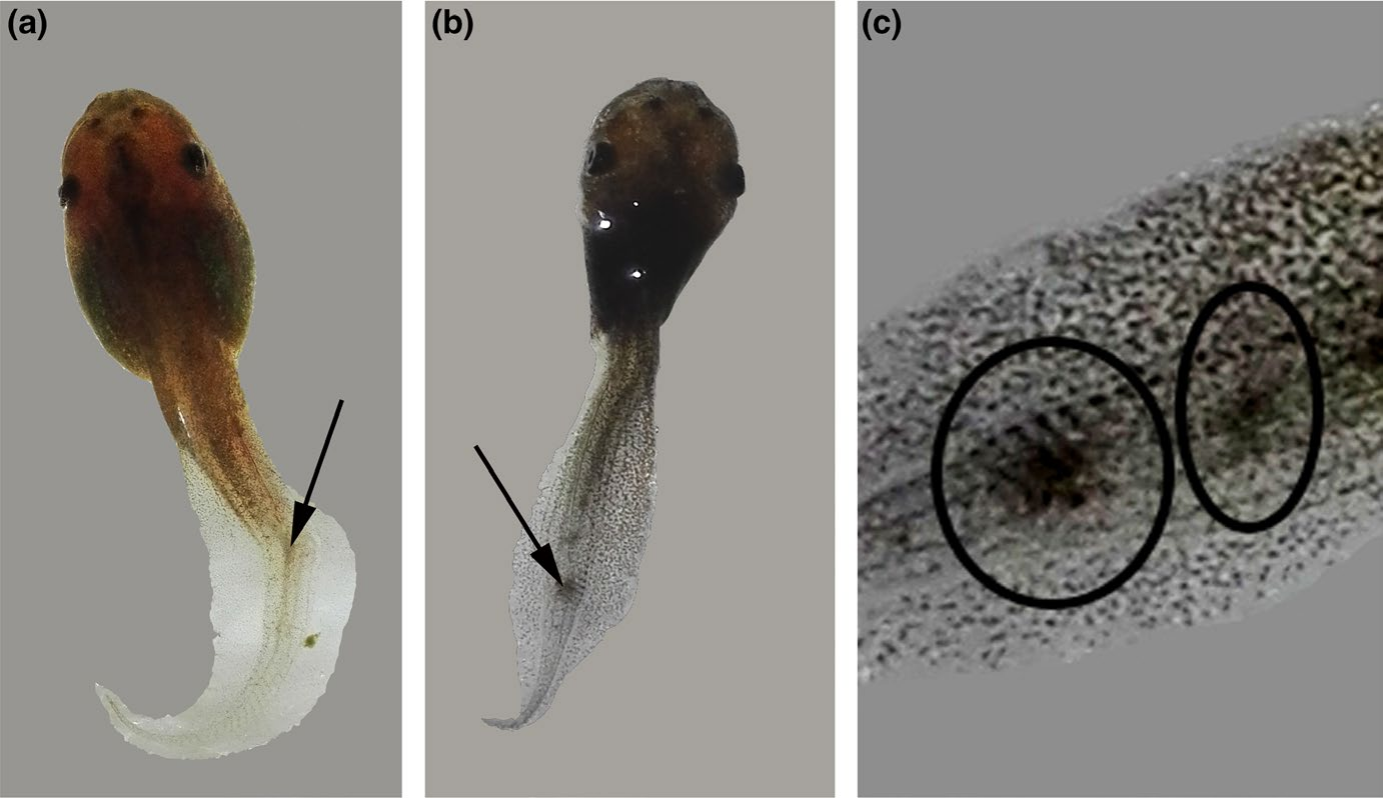
Metacercarial cysts inside the tail of tadpoles of *Polypedates cruciger* exposed to the two cercaria types, pleurolophocercous cercaria of *Acanthostomum burminis* and furcocercous cercaria (a, b), enlarged metacercarial cysts (c)

**Figure 3:**
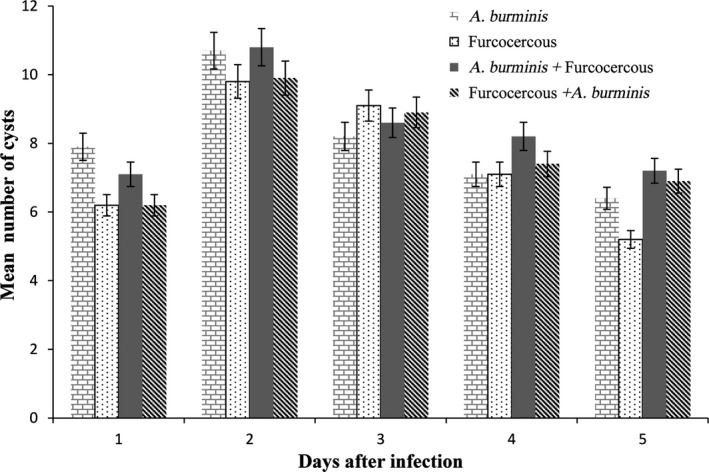
Mean number of cysts in tadpoles of *Polypedates cruciger* for five consecutive days postexposure. No cysts were observed in the tadpoles in the control group

Most of the cercaria of both types penetrated into the tail region of the tadpoles, and the rest penetrated the other parts of the body such as head and eyes. The metacercarial cysts of the cercaria that penetrated the tail region were visible through the dissecting microscope (Figure 2). However, it was not possible to distinguish the cysts of the two cercaria types. A similar percentage of cercaria penetrated the tail region of the tadpoles (*A. burminis* = 83.9%, furcocercous = 79.3%, *A. burminis* + furcocercous = 83.2%, furcocercous + *A. burminis* = 78.7%). An increase in the average number of cysts was observed for all treatments until the second day, after which the numbers decreased as some cysts disappeared (Figure 3). There was no difference in the disappearing of cysts in the tadpoles exposed to *A. burminis* and furcocercous (one‐way ANOVA; *F *= 0.32, *p = *0.590; Figure 3) or between the two combined exposures (one‐way ANOVA, *F *= 0.28, *p = *0.614; Figure 3). There was no difference in the number of cysts in lone infection and coinfection (one‐way ANOVA, *p* > 0.05). There were no cysts in the tadpoles in the control group.

### 3.5 Growth of the tadpoles and metermorphs

There was no size difference among the tadpoles in different treatments (one‐way ANOVA, *p* > 0.05; Table 3). However, differences in the length of the developmental period were observed in some exposures (one‐way ANOVA, *p* < 0.05; Table 3). Tadpoles exposed to *A. burminis* (lone infection) and the two coinfections showed a significant lengthening of growth period compared to the overall control but not those exposed to furcocercous (one‐way ANOVA, Dunnett's post hoc test; Table 3). There was no difference in the growth period between lone infection of furcocercous and coinfections (one‐way ANOVA; *F* = 1.29, *p* = 0.297).

**Table 3:** Growth of the tadpoles measured as TE_50 _(time required for forelimb emergence of half of the number of tadpoles), snout to vent length (SVL), and body mass of *Polypedates cruciger* exposed to cercaria of *Acanthostomum burminis* (cercaria P) and furcocercous (cercaria F) cercaria at 10 days posthatch using one‐way ANOVA and Dunnett's post hoc test

Treatment	*F*		*p*
Growth period			
Mean TE_50_ ± *SD* (days)			
Control	97.2 ± 7.8		
Cercaria P only	111.3 ± 14.1	4.37	0.019**
Cercaria F only	105.4 ± 9.3		0.285
Cercaria P + cercaria F	115.7 ± 8.9		0.002*
Cercaria F + cercaria P	109.4 ± 9.9		0.043**
Length			
Mean SVL ± *SD *(cm)			
Control	2.68 ± 0.08	–	–
Cercaria P only	2.65 ± 0.05	0.53	0.716***
Cercaria F only	2.65 ± 0.08		
Cercaria P + cercaria F	2.66 ± 0.07		
Cercaria F + cercaria P	2.68 ± 0.08		
Weight			
Mean weight ± *SD* (g)			
Control	0.707 ± 0.052		
Cercaria P only	0.697 ± 0.044	0.26	0.903***
Cercaria F only	0.710 ± 0.045		
Cercaria P + cercaria F	0.698 ± 0.036		
Cercaria F + cercaria P	0.705 ± 0.040		

* Significant differences at *p* < 0.001.

** Significant.

*** Not significant differences at *p* < 0.05 analyzed using one‐way ANOVA and Dunnett's post hoc test for individual comparisons, and the rest were not significant compared to the control. Multiple *p* values indicate the results of the post hoc test for significant means. *n* = 40 tadpoles in each treatment and 40 in the control.

### 3.6 Malformations in tadpoles and metermorphs

**Figure 4:**
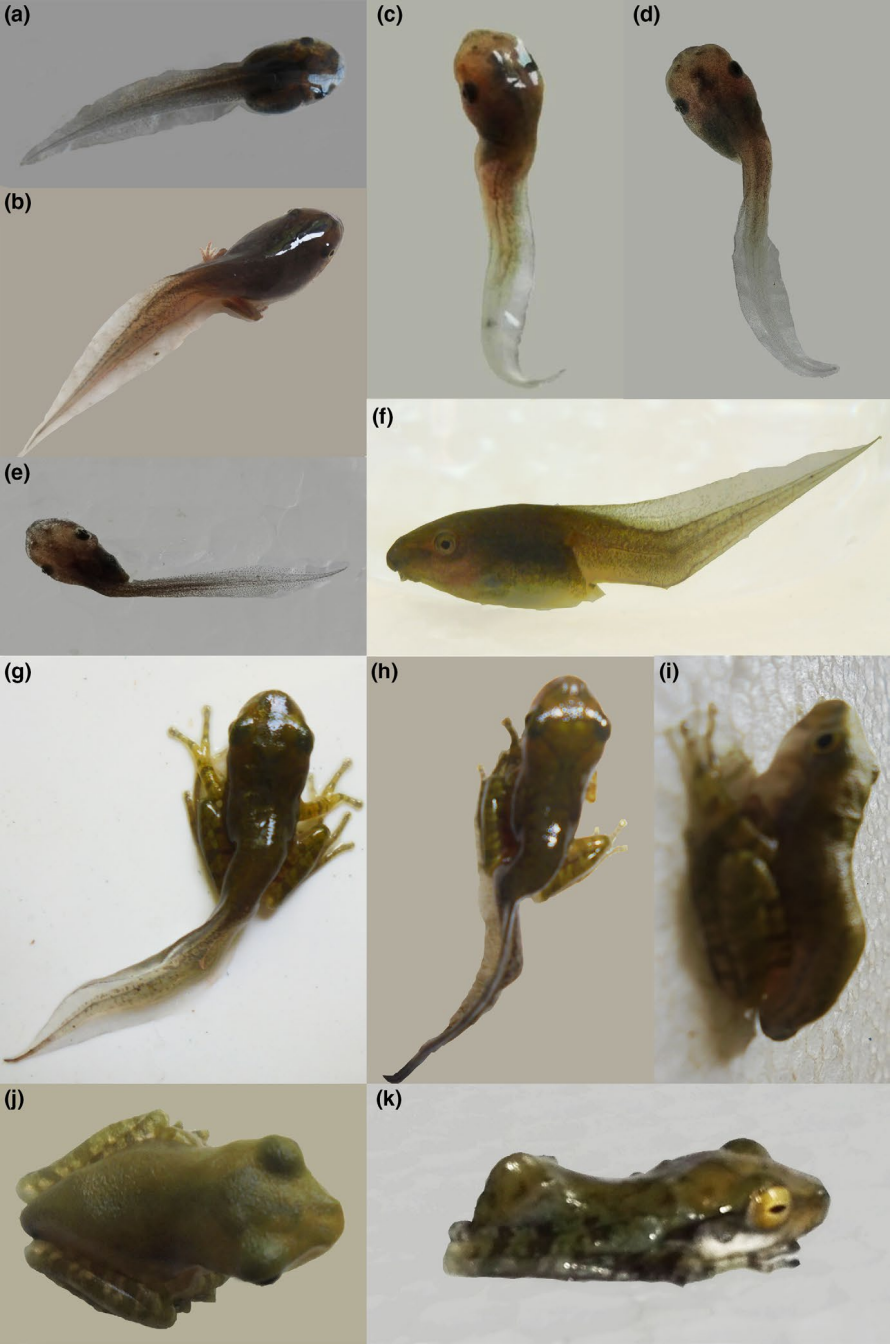
Representative malformations in the spine of tadpoles and metamorphs of *Polypedates cruciger* exposed to cercaria. (a) Normal tadpole, (b–d, h, i) scoliosis (lateral deviation in the normally straight line of the spine), (j) normal metermorph, (e–g, k) kyphosis (hunched back/curvature of the spine in the dorsoventral plane)

Exposed tadpoles in all treatments developed malformations. Scoliosis (lateral deviation of the spine) and kyphosis (deviation of spine in the dorsal and ventral plane) were the most common types (Figure 4). Malformations were absent in the tadpoles in the control group. There was no difference in the types of malformation and the percentage of malformed individuals in lone exposures or coinfections (percentage malformations at 40 days posthatch; *A. burminis* = 57.5%, furcocercous = 48.0%, *A. burminis* + furcocercous = 52.5%, furcocercous + *A. burminis* = 52.5%; Table 4). Dead tadpoles with malformations were also considered for the percentage calculations. Among the two types, scoliosis was the most observed malformation in all cercaria treatment at early stages. However, tadpoles with scoliosis in the tail did not show any signs of scoliosis as metamorphs (Table 5).

**Table 4:** Malformations at 40 days posthatch tadpoles (Gosner stage 31/32) and metamorphs of *Polypedates cruciger* exposed to cercaria (*Acanthostomum burminis*—cercaria P and furcocercous—cercaria F) treatments using binomial generalized linear model (GLM) test

Treatment	40 days posthatch					Metermorphs				
	Malformation (%)	Comparison with cercaria P only		Comparison with cercaria F only		Malformation (%)	Comparison with cercaria P only		Comparison with cercaria F only	
		*F*	*p*	*F*	*p*		*F*	*p*	*F*	*p*
Cercaria P + F	52.5	0.20	0.658	0.05	0.826	22.5	0.07	0.796	0.07	0.788
Cercaria F + P	52.5	0.20	0.658	0.05	0.826	20.0	0.28	0.598	0.08	0.778

Cercaria P = *A. burminis* and cercaria *F* = furcocercous; percentage malformations for cercaria P were 57.5% and 25.0% and cercaria F were 48.0% and 19.0% for the tadpoles at 40 days posthatch and metamorphs, respectively. *n* = 40 tadpoles in each treatment and 40 in the control.

**Table 5:** Types of malformations in tadpoles at 40 days posthatch and in metamorphs of *Polypedates cruciger* exposed to two cercaria types (*Acanthostomum burminis*—Cercaria P and furcocercous—cercaria F)

Percentage of malformed individuals (%)								
Malformation type	Cercaria P only		Cercaria F only		Cercaria P + cercaria F		Cercaria F + cercaria P	
	40 days posthatch	Metamorphs	40 days posthatch	Metamorphs	40 days posthatch	Metamorphs	40 days posthatch	Metamorphs
Kyphosis	25.0	25.0	20.0	19.0	22.5	22.5	22.5	20.0
Scoliosis	32.5	0.0	27.5	0.0	30.0	0.0	30.0	0.0

The percentages were calculated by dividing the number of malformed individuals from the initial number (40 in each treatment and control) of individuals exposed to each cercaria type at 40 days posthatch stage and at metamorphosis. Malformations in the dead tadpoles were also considered.

## 4 DISCUSSION

The sequence of exposure in the coinfection of two larval trematodes was associated with the disease severity of the amphibian host, *P. cruciger*. When the cercaria of *A. burminis* were introduced prior to furcocercous cercaria, infection severity was increased while infection with furcocercous prior to *A. burminis* resulted in reduced infection severity on the host. There was no difference in the infection success, but the survival was decreased by 17.0%. This shows that when exposures of the two parasites are temporally staggered, the sequence of addition has an effect on the disease outcome. These results are consistent with the findings of Hoverman et al. (2013), where they report *Ribeiroia* infection success in the Pacific chorus frog, *Pseudacris regilla*, is reduced when *Echinostoma* is added prior to *Ribeiroia*. Moreover, a recent study shows coinfection priority effects in an amphibian–trematode–virus disease system (Wuerthner, Hua, & Hoverman, 2017). These authors have shown that *Echinoparyphium* infections can be beneficial for hosts challenged with ranavirus provided that ranavirus exposure occurs later in development and they conclude that temporal separation between parasite exposures could be a function of changes in host traits that influence susceptibility (Wuerthner et al., 2017).

The survival of tadpoles in the lone infections was lower compared to that of the coinfection of furcocercous prior to *A. burminis* but higher in the coinfection of *A. burminis* prior to furcocercous. Higher host survival could be beneficial for transmission of furcocercous if *A. burminis* exposure occurs later. Interactions among coinfection of more than one parasites can affect host pathology, parasite transmission, and virulence evolution (Rigaud et al., 2010), while abiotic environmental stressors can strongly influence susceptibility to disease in amphibians (Rohr et al., 2008) and might control whether interactions between pathogens occur in nature.

Infection of cercaria of *A. burminis,* to the hourglass tree frog, *P. cruciger*, and the Asian common toad *Duttaphrynus melanostictus* showed that it induces axial and some limb malformations, increasing mortality and time to metamorphosis, while reducing size at metamorphosis (Jayawardena, Rajakaruna, Navaratne, et al., 2010b; Jayawardena, Tkach, Navaratne, Amerasinghe, & Rajakaruna, 2013; Rajakaruna et al., 2008); this study also corroborates this. However, not all cercaria induce significant negative effects on amphibian host. Exposure to nine types of cercaria to *P. cruciger* showed only three types lower survival, lengthen growth period, and contribution to development of malformations (Pathirana & Rajakaruna, 2018).

This study adds another cercaria type, furcocercous cercaria, to the list of cercaria that induce malformations in local amphibians. Furcocercous cercaria successfully penetrated the tadpoles of *P. cruciger* and reduced survival and induced malformations, which were mainly kyphosis and scoliosis, which were similar to those induced by *A. burminis* cercaria on the same host (Rajakaruna et al., 2008). Exposure however did not affect the developmental period of tadpoles as was apparent in *A. burminis*‐infected tadpoles (Jayawardena, Rajakaruna, Navaratne, et al., 2010b; Rajakaruna et al., 2008). Most of the malformations were in the tail of the tadpoles, and when the tail was absorbed during metamorphosis, these malformations disappeared, and hence, fewer metamorphs compared to tadpoles had malformations.

The metacercaria encystment and the formation of cysts were visible under the dissecting microscope due to lack of skin pigmentation in *P. cruciger* tadpoles. An increase in the number of cysts was observed for all the treatments in day 2, but thereafter, the numbers decreased. The reason for the initial increase in the number of metacercarial cysts is unknown but it could be that some of the cysts that were being formed may have been missed during the initial counts as the first counting was done 30 min after exposure. The reduction of the cysts could be due to the immunological reactions of the host toward the parasite, destroying and absorbing the cysts (Stopper, Hecker, Franssen, & Sessions, 2002). Moreover, some of the cysts disappeared when the tail of the tadpole was absorbed during metamorphosis. Both cercaria induced axial malformations. The mechanism of inducing the axial malformations could be because of the cyst disturbing the arrangement of vertebral column by chemical or physical disturbances acting independently or in concert (Johnson et al., 2002).

When hosts are exposed to parasites, they mount immune response, which is energetically expensive (Sheldon & Verhulst, 1996). Studies have shown that the cost of immune response alters the resource allocation in growth (Martin, 2005) and reproduction (Bonneaud et al., 2003; Uller, Isaksson, & Olsson, 2006). Moreover, activation of one immune response can negatively affect a second immune response (Martin, Weil, Kuhlman, & Nelson, 2006). Both cercaria encysted in the same region of the tadpole body, mostly in the tail. When the parasites share same site of encystment, there may be high resource demands and competitive interactions (Smyth & Halton, 1983). Studies have shown that the effect of single parasite is more compared to coinfections when the two parasites’ site of encystment is different, and therefore, negative interactions are mediated by the immune system. For example, coinfection of cercaria that encyst in different locations such as *Echinostoma* which encysts in kidney and *Ribeiroia* in limb buds is known to have low resource demands, and the competitive interactions are mediated by the host immune system (Hoverman et al., 2013; Johnson & Hoverman, 2012). However, in the present study both parasites encyst in the same location, and therefore, coinfection had more profound effects (high resource demands), which may not be well mediated by the immune system.

Parasite transmission in natural populations is a dynamic process, influenced by spatial and temporal heterogeneity in the abundance of both hosts and parasites (Hawley & Altizer, 2011). Moreover, the ability of hosts to resist or tolerate infection can vary temporally, such as between seasons (Hawley & Altizer, 2011) or through development (Holland et al., 2007; Johnson, Kellermanns, & Bowerman, 2011; Kelly, Thomas, Thieltges, Poulin, & Tompkins, 2010). These factors may modulate potential direct and indirect interactions among parasites within hosts, suggesting that priority effects could be important for structuring intrahost–parasite communities (Jackson et al., 2006; Karvonen et al., 2009). Understanding parasite interactions and the burden of the effect within hosts would be a key factor in host disease outcome and hence in amphibian conservation.

## CONFLICT OF INTEREST

None declared.

## AUTHOR CONTRIBUTION

NUKP and RR conceived the idea and designed methodology. NUKP collected and analyzed data, and wrote the manuscript. RSR and MM critically edited the manuscript.

## DATA AVAILABILITY STATEMENT

Data are available from the Dryad Digital Repository, https://doi.org/10.5061/dryad.60j73s7.

## References

[ece35180-bib-0001] Abbas, A. K. , Murphy, K. M. , & Sher, A. (1996). Functional diversity of helper T lymphocytes. Nature, 383, 787–793. 10.1038/383787a0 8893001

[ece35180-bib-0002] Alford, R. A. , & Wilbur, H. M. (1985). Priority effects in experimental pond communities: Competition between *Bufo* and *Rana* . Ecology, 66, 1097–1105. 10.2307/1939161

[ece35180-bib-0003] Bentwich, Z. , Kalinkovich, A. , Weisman, Z. , Borkow, G. , Beyers, N. , & Beyers, A. D. (1999). Can eradication of helminthic infections change the face of AIDS and tuberculosis? Immunology Today, 20, 485–487. 10.1016/S0167-5699(99)01499-1 10529774

[ece35180-bib-0004] Bonneaud, C. , Mazuc, J. , Gonzalez, G. , Haussy, C. , Chastel, O. , Faivre, B. , & Sorci, G. (2003). Assessing the cost of mounting an immune response. American Naturalist, 161, 367–379. 10.1086/346134 12703483

[ece35180-bib-0005] Cobey, S. , & Lipsitch, M. (2013). Pathogen diversity and hidden regimes of apparent competition. American Naturalist, 181, 12–24. 10.1086/668598 PMC3716377 23234842

[ece35180-bib-0006] Connell, J. H. , & Slatyer, R. O. (1977). Mechanisms of succession in natural communities and their role in community stability and organization. American Naturalist, 111, 1119–1144. 10.1086/283241

[ece35180-bib-0007] Cox, T. (2001). Creating the multicultural organization: A strategy for capturing the power of diversity. University of Michigan Business School Management Series.

[ece35180-bib-0008] Cunningham, A. A. , Langton, T. E. S. , Bennett, P. M. , Lewin, J. F. , Drury, S. E. N. , Gough, R. E. , & Macgregor, S. K. (1996). Pathological and microbiological findings from incidents of unusual mortality of the Common Frog (*Rana temporaria*). Philosophical Transactions of the Royal Society of London. Series B, Biological Sciences, 351, 1539–1557. 10.1098/rstb.1996.0140 8962441

[ece35180-bib-0009] Daszak, P. , Cunningham, A. A. , & Hyatt, A. D. (2003). Infectious disease and amphibian population declines. Diversity and Distributions, 9, 141–150. 10.1046/j.1472-4642.2003.00016.x

[ece35180-bib-0010] Green, D. E. , Converse, K. A. , & Schrader, A. K. (2002). Epizootiology of sixty‐four amphibian morbidity and mortality events in the USA, 1996–2001. Annals of the New York Academy of Sciences, 969, 323–339. 10.1111/j.1749-6632.2002.tb04400.x 12381613

[ece35180-bib-0011] Greer, A. L. , Berrill, M. , & Wilson, P. J. (2005). Five amphibian mortality events associated with ranavirus infection in south central Ontario, Canada. Diseases of Aquatic Organisms, 67, 9–14. 10.3354/dao067009 16385802

[ece35180-bib-0012] Hawley, D. M. , & Altizer, S. M. (2011). Disease ecology meets ecological immunology: Understanding the links between organismal immunity and infection dynamics in natural populations. Functional Ecology, 25, 1471–1480. 10.1111/j.1365-2435.2010.01753.x

[ece35180-bib-0013] Holland, M. P. , Skelly, D. K. , Kashgarian, M. , Bolden, S. R. , Harrison, L. M. , & Cappello, M. (2007). Echinostome infection in green frogs (*Rana clamitans*) is stage and age dependent. Journal of Zoology, 271, 455–462. 10.1111/j.1469-7998.2006.00229.x

[ece35180-bib-0014] Hoverman, J. T. , Hoye, B. J. , & Johnson, P. T. J. (2013). Does timing matter? How priority effects influence the outcome of parasite interactions within hosts. Oecologia, 173, 1471–1480. 10.1007/s00442-013-2692-x 23754306

[ece35180-bib-0015] Hoverman, J. T. , Mihaljevic, J. R. , Richgels, K. L. D. , Kerby, J. L. , & Johnson, P. T. J. (2012). Widespread co‐occurrence of virulent pathogens within California amphibian communities. EcoHealth, 9, 288–292. 10.1007/s10393-012-0778-2 22766887

[ece35180-bib-0016] Huffman, J. E. , & Fried, B. (2012). The biology of *Echinoparyphium* (Trematoda, Echinostomatidae). Acta Parasitologica, 57, 199–210. 10.2478/s11686-012-0042-5 22875668

[ece35180-bib-0017] IUCN Red list for threatened species . (2018). Species range. Retrieved from: http://www.iucnredlist.org/details/58943/0

[ece35180-bib-0018] Jackson, J. A. , Pleass, R. J. , Cable, J. , Bradley, J. E. , & Tinsley, R. C. (2006). Heterogenous interspecific interactions in a host–parasite system. International Journal for Parasitology, 36, 1341–1349. 10.1016/j.ijpara.2006.07.003 16934815

[ece35180-bib-0019] Jayawardena, U. A. , Rajakaruna, R. S. , & Amerasinghe, P. H. (2010a). Cercariae of trematodes in freshwater snails in three climatic zones in Sri Lanka. Ceylon Journal of Science (Biological Sciences), 39, 95–108. 10.4038/cjsbs.v39i2.2996

[ece35180-bib-0020] Jayawardena, U. A. , Rajakaruna, R. S. , Navaratne, A. N. , & Amerasinghe, P. H. (2010b). Monostome cercariae induced malformations in amphibians: Effect of infection at the pre‐limb‐bud stage tadpoles of *Polypedates cruciger* Blyth. Journal of the National Science Foundation of Sri Lanka, 38, 241–248.

[ece35180-bib-0021] Jayawardena, U. A. , Tkach, V. V. , Navaratne, A. N. , Amerasinghe, P. H. , & Rajakaruna, R. S. (2013). Malformations and mortality in the Asian Common Toad induced by exposure to pleurolophocercous cercariae (Trematoda: Cryptogonimidae). Parasitology International, 62, 246–252. 10.1016/j.parint.2013.01.003 23353759

[ece35180-bib-0022] Johnson, P. T. J. , & Buller, I. D. (2011). Parasite competition hidden by correlated coinfection: Using surveys and experiments to understand parasite interactions. Ecology, 92, 535–541. 10.1890/10-0570.1 21608460

[ece35180-bib-0023] Johnson, P. T. J. , & Hoverman, J. T. (2012). Parasite diversity and coinfection determine pathogen infection success and host fitness. Proceedings of the National Academy of Sciences, 109, 9006–9011. 10.1073/pnas.1201790109 PMC3384156 22615371

[ece35180-bib-0024] Johnson, P. T. J. , Kellermanns, E. , & Bowerman, J. (2011). Critical windows of disease risk: Amphibian pathology driven by developmental changes in host resistance and tolerance. Functional Ecology, 25, 726–734. 10.1111/j.1365-2435.2010.01830.x

[ece35180-bib-0025] Johnson, P. T. J. , Lunde, K. B. , Ritchie, E. G. , & Launer, A. E. (1999). The effect of trematode infection on amphibian limb development and survivorship. Science, 284, 802–804. 10.1126/science.284.5415.802. 10221912

[ece35180-bib-0026] Johnson, P. T. J. , Lunde, K. B. , Thurman, E. M. , Ritchie, E. G. , Wray, S. N. , Sutherland, D. R. , … Blaustein, A. R. (2002). Parasite (*Ribeiroia ondatrae*) infection linked to amphibian malformations in the western United States. Ecological Monographs, 15, 34–35. 10.1890/0012-9615(2002)072[0151:PROILT]2.0.CO;2

[ece35180-bib-0027] Jolles, A. E. , Ezenwa, V. O. , Etienne, R. S. , Turner, W. C. , & Olff, H. (2008). Interactions between macroparasites and microparasites drive infection patterns in free‐ranging African buffalo. Ecology, 89, 2239–2250. 10.1890/07-0995.1 18724734

[ece35180-bib-0028] Karvonen, A. , Seppälä, O. , & Tellervo Valtonen, E. (2009). Host immunization shapes interspecific associations in trematode parasites. Journal of Animal Ecology, 78, 945–952. 10.1111/j.1365-2656.2009.01562.x 19457020

[ece35180-bib-0029] Kelly, D. W. , Thomas, H. , Thieltges, D. W. , Poulin, R. , & Tompkins, D. M. (2010). Trematode infection causes malformations and population effects in a declining New Zealand fish. Journal of Animal Ecology, 79, 445–452. 10.1111/j.1365-2656.2009.01636.x 19886894

[ece35180-bib-0030] Koprivnikar, J. , Marcogliese, D. J. , Rohr, J. R. , Orlofske, S. A. , Raffel, T. R. , & Johnson, P. T. J. (2012). Macroparasite infections of amphibians: What can they tell us? EcoHealth, 9, 342–360. 10.1007/s10393-012-0785-3 22810498

[ece35180-bib-0031] Leung, T. L. F. , & Poulin, R. (2011). Intra‐host competition between co‐infecting digeneans within a bivalve second intermediate host: Dominance by priority‐effect or taking advantage of others? International Journal for Parasitology, 41, 449–454. 10.1016/j.ijpara.2010.11.004 21167832

[ece35180-bib-0032] Lively, C. M. (2009). Local host competition in the evolution of virulence. Journal of Evolutionary Biology, 22, 1268–1274. 10.1111/j.1420-9101.2009.01743.x 19490389

[ece35180-bib-0033] López‐Villavicencio, M. , Courjol, F. , Gibson, A. K. , Hood, M. E. , Jonot, O. , Shykoff, J. A. , & Giraud, T. (2011). Competition, cooperation among kin, and virulence in multiple infections. Evolution 65, 1357–1366. 10.1111/j.1558-5646.2010.01207.x 21121914

[ece35180-bib-0034] Manamendra‐Arachci , K. , & Dutta , S. K. (1996). The Amphibian Fauna of Sri Lanka. Colombo, Sri Lanka: Wildlife Heritage Trust of Sri Lanka.

[ece35180-bib-0035] Martin, L. B. II (2005). Trade‐offs between molt and immune activity in two populations of house sparrows (*Passer domesticus*). Canadian Journal of Zoology, 83, 780–787. 10.1139/z05-062

[ece35180-bib-0036] Martin, L. B. II , Weil, Z. M. , Kuhlman, J. R. , & Nelson, R. J. (2006). Trade‐offs within the immune systems of female White‐footed Mice, *Peromyscus leucopus* . Functional Ecology, 20, 630–636. 10.1111/j.1365-2435.2006.01138.x

[ece35180-bib-0037] Meteyer, C. U. (2000). Field guide to malformations of frogs and toads with radiographic interpretations. USGS National Wildlife Health Centre. Downloaded on 04 April 2017.

[ece35180-bib-0038] Pathirana, N. U. K. , Meegaskumbura, M. , & Rajakaruna, R. S. (2016). Age dependent tolerance to parasitism: Trematode infections in tadpoles of common hourglass tree frog (*Polypedates cruciger*). Peradeniya University International Research Sessions iPURSE.20:299. 10.13140/RG.2.2.23076.53127.

[ece35180-bib-0039] Pathirana, N. U. K. , Meegaskumbura, M. , & Rajakaruna, R. S. (2017). Host resistance to parasitism: Do older tadpoles of *Polypedates cruciger* show higher resistance towards pleurolophocercous cercariae? International Forestry and Environment Symposium, University of Sri Jayawardenapura.22:22. 10.31357/fesympo.v22i0.3268.

[ece35180-bib-0040] Pathirana, N. U. K. , & Rajakaruna, R. S. (2018). Trematode infections in frogs: Do all cercarial morphotypes infect and induce effects on the common hourglass tree frog, *Polypedates cruciger*? Ceylon Journal of Science (Biological Sciences), 47, 319–330. doi: 10.4038/cjs.v47i4.7549

[ece35180-bib-0041] Pedersen, A. B. , & Babayan, S. A. (2011). Wild immunology. Molecular Ecology, 22, 133–139. 10.1111/j.1365-294X.2010.04938.x 21324009

[ece35180-bib-0042] Pedersen, A. B. , & Fenton, A. (2007). Emphasizing the ecology in parasite community ecology. Trends in Ecology & Evolution, 22, 133–139. 10.1016/j.tree.2006.11.005 17137676

[ece35180-bib-0043] Petney, T. N. , & Andrews, R. H. (1998). Multiparasite communities in animals and humans: Frequency, structure and pathogenic significance. International Journal for Parasitology, 28, 377–393. 10.1016/S0020-7519(97)00189-6 9559357

[ece35180-bib-0044] Rajakaruna, R. S. , Piyatissa, P. M. J. R. , Jayawardena, U. A. , Navaratne, A. N. , & Amerasinghe, P. H. (2008). Trematode infection induced malformations in the common hourglass treefrogs. Journal of Zoology, 275, 89–95. 10.1111/j.1469-7998.2008.00416.x

[ece35180-bib-0045] Rigaud, T. , Perrot‐Minnot, M.‐J. , Brown, M. J. F. , Ahonen, R. , Puustinen, S. , Mutikainen, P. , … Haydon, D. T. (2010). Parasite and host assemblages: embracing the reality will improve our knowledge of parasite transmission and virulence. Proceedings of the Royal Society B: Biological Sciences 277, 3693–3702. 10.1098/rspb.2010.1163. PMC2992712 20667874

[ece35180-bib-0046] Rohr, J. R. , Raffel, T. R. , & Hall, C. A. (2010). Developmental variation in resistance and tolerance in a multi‐host‐parasite system. Functional Ecology, 24, 1110–1121. 10.1111/j.1365-2435.2010.01709.x

[ece35180-bib-0047] Rohr, J. R. , Schotthoefer, A. M. , Raffel, T. R. , Carrick, H. J. , Halstead, N. , Hoverman, J. T. , … Beasley, V. R. (2008). Agrochemicals increase trematode infections in a declining amphibian species. Nature, 455, 1235–1239. 10.1038/nature07281 18972018

[ece35180-bib-0048] Romansic, J. M. , Johnson, P. T. J. , Searle, C. L. , Johnson, J. E. , Tunstall, T. S. , Han, B. A. , … Blaustein, A. R. (2011). Individual and combined effects of multiple pathogens on Pacific treefrogs. Oecologia, 166, 1029–1041. 10.1007/s00442-011-1932-1 21400194

[ece35180-bib-0049] Sheldon, B. C. , & Verhulst, S. (1996). Ecological immunology: Costly parasite defences and trade‐offs in evolutionary ecology. Trends in Ecology & Evolution, 11, 317–321. 10.1016/0169-5347(96)10039-2 21237861

[ece35180-bib-0050] Smyth, J. D. , & Halton, D. W. (1983). The physiology of trematodes. Cambridge, UK: Cambridge University Press.

[ece35180-bib-0051] Stopper, G. F. , Hecker, L. , Franssen, R. A. , & Sessions, S. K. (2002). How trematodes cause limb deformities in amphibians. Journal of Experimental Zoology, 294, 252–263. 10.1002/jez.10173 12362431

[ece35180-bib-0052] Telfer, S. , Birtles, R. , Bennett, M. , Lambin, X. , Paterson, S. , & Begon, M. (2008). Parasite interactions in natural populations: Insights from longitudinal data. Parasitology, 135, 767–781. 10.1017/S0031182008000395 18474121 PMC2952918

[ece35180-bib-0053] Telfer, S. , Lambin, X. , Birtles, R. , Beldomenico, P. , Burthe, S. , Paterson, S. , & Begon, M. (2010). Species interactions in a parasite community drive infection risk in a wildlife population. Science, 330, 243–246. 10.1126/science.1190333 20929776 PMC3033556

[ece35180-bib-0054] Uller, T. , Isaksson, C. , & Olsson, M. (2006). Immune challenge reduces reproductive output and growth in a lizard. Functional Ecology, 20, 873–879. 10.1111/j.1365-2435.2006.01163.x

[ece35180-bib-0055] Warne, R. W. , LaBumbard, B. , LaGrange, S. , Vredenburg, V. T. , & Catenazzi, A. (2016). Co‐infection by Chytrid fungus and Ranaviruses in wild and harvested frogs in the Tropical Andes. PLoS ONE, 11, e0145864 10.1371/journal.pone.0145864 26726999 PMC4701007

[ece35180-bib-0056] Worthylake, K. M. , & Hovingh, P. (1989). Mass mortality of salamanders (*Ambystoma tigrinum*) by bacteria (Acinetobacter) in an oligotrophic seepage mountain lake. Great Basin Naturalist, 49, 364–372. 10.2307/41712656

[ece35180-bib-0057] Wuerthner, V. P. , Hua, J. , & Hoverman, J. T. (2017). The benefits of coinfection: Trematodes alter disease outcomes associated with virus infection. Journal of Animal Ecology, 86, 921–931. 10.1111/1365-2656.12665 28317105

